# Antimicrobial Resistance in Nepal

**DOI:** 10.3389/fmed.2019.00105

**Published:** 2019-05-24

**Authors:** Krishna Prasad Acharya, R. Trevor Wilson

**Affiliations:** ^1^Ministry of Land Management, Agriculture and Co-operatives (MoLMAC), Gandaki, Nepal; ^2^Regional Veterinary Laboratory, Pokhara, Nepal; ^3^Bartridge House, Umberleigh, United Kingdom

**Keywords:** antimicrobial resistance, public health, antibiotic residues, animal health, Nepal

## Abstract

Antimicrobial resistance (AMR) is a global problem to animal and public health. It has drawn the attention of public health experts, stakeholders, and medical science due to the substantial economic loss that it causes to individuals and nation as a whole. Various cross-sectional studies and some national surveys in developing countries have shown increase in the burden of antimicrobial resistance. Nepal is one of the major contributors to the growing burden of AMR due to widespread irrational use of antibiotics along with poor health care systems poor infection control and prevention measures. This review was conducted to summarize the situation of AMR in Nepal, determinants of AMR, current government intervention strategies and the way forward to reduce the AMR burden in Nepal. Available cross sectional reports warn that bacterial pathogens are becoming highly resistant to most first- and some second-line antibiotics. The irrational and injudicious use of high doses of antibiotics for therapy and sub-optimal doses as growth promoters are leading causes of AMR in Nepal. Establishment of a surveillance programme and a national plan for containment of AMR, following the National Antibiotics Treatment Guideline 2014 and generation of awareness among veterinarians, technicians, and medical physicians on prudent use of antimicrobial drugs in Nepal could reduce the burden of AMR. In addition, there is a need to develop a national laboratory strategic plan to provide guidance and governance to national laboratories.

## Introduction

**Figure 1:**
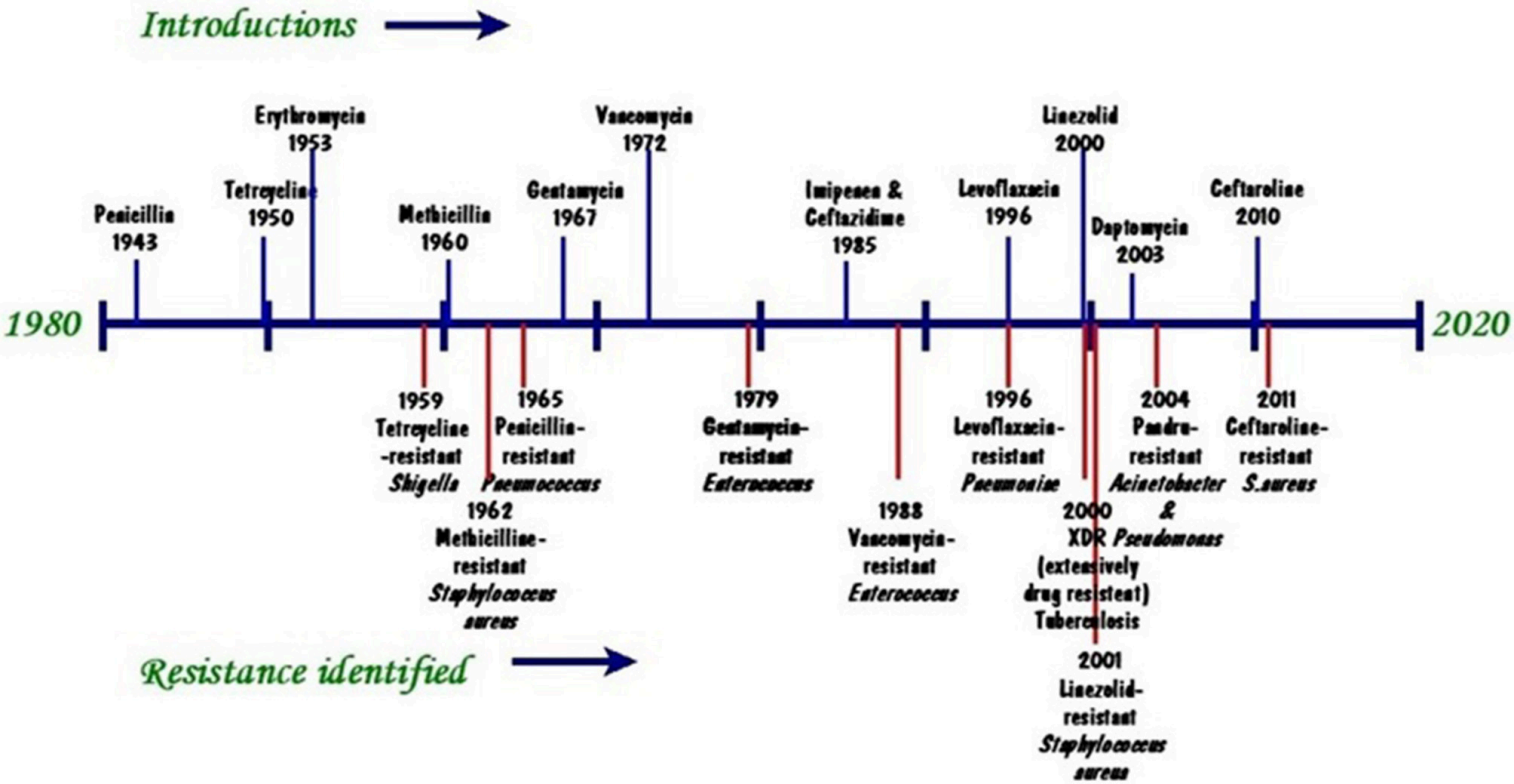
Timeline of key antibiotic resistance events (Source: modified from CDC 2013. https://www.cdc.gov/drugresistance/pdf/ar-threats-2013-508.pdf).

Antibiotics were considered a miracle cure against disease and for prolonging life. Penicillin was the first antibiotic used by man to save millions of people's lives during and after World War Two. Since then the use of antibiotics has expanded exponentially (1) and has resulted in antimicrobial resistance (AMR). The major factor in increasing AMR in the world is the indiscriminate, inappropriate, and inadequate use of antibiotics. In developing countries antimicrobial resistance is driven by the high incidence of infectious diseases (2), inappropriate use of antibiotics in treatment (3), use of antibiotics as growth promoters (4) and lack or poor implementation of legislation to AMR (5). AMR is, nevertheless, a global issue of public health concern. For some 30 years no new antibiotics have been discovered whilst existing ones fail to suppress or kill the target microorganisms (6). The trend of antibiotic resistance development is illustrated by a recent worldwide analysis (Figure 1). An emerging group of antibiotic resistant bacteria, denominated ESKAPE (*Enterococcus faecium, Staphylococcus aureus, Klebsiella pneumoniae, Acinetobacter baumanni, Pseudomonas aeruginosa*, and *Enterobacter* spp.) in humans (1, 7–9) and *E. coli, Salmonella spp., Staphylococcus spp., Streptocccus spp., Pasteurella spp.*, and *Pseudomonas spp*. in animals (1, 7, 10) has been identified as being able to escape the action of antibiotics and represents new paradigms in pathogenesis, transmission, and resistance. The situation has been further worsened by the rapid increase in resistant microbes compounded by the lack of discovery of new highly effective antibiotics (11).

Nepal is a Himalayan country located between the two major emerging economies of China and India. Nepal's economy, where 27.8 million people live, is primarily based on agriculture and livestock. The livestock sector alone contributes around 13% to the national gross domestic products (GDP) and over a quarter (27%) to the agricultural gross domestic product (AGDP) (12). The demand for livestock products has increased with increasing per caput income and hence poultry, pig, and dairy industries are also expanding rapidly. Livestock farming is growing at an annual rate of around 1.23% in Nepal (13). With modernization, livestock production is shifting toward intensification and away from traditional subsistence farming (5, 14). Concurrently the use of antimicrobials has skyrocketed to achieve a faster growth rate and to prevent or treat diseases (5, 15).

Factors such as the use of antibiotics as growth promoter in animal agriculture, overuse, use for long periods, self-medication practices and lack of well-equipped hospitals and clinics have been drivers of the development of AMR in humans (1, 16) whereas factors such as poor husbandry practices with inappropriate infection prevention and control (IPC), lack of awareness on good management practices (GMP) and prudential use of antibiotics have contributed to development of AMR in the animal health sector (1).

In Nepal, AMR is often neglected owing to several other public health priorities and improper implementation of the law (5). The current practice of common antimicrobials use in people and animals along with the problem of antimicrobial residue and resistance is an alarm bell for an even worse public health situation (5, 14, 15). This paper provides a review of antimicrobial resistance, determinants of AMR, current government strategies to tackle it and a way forward for prudent use of antibiotics in people and animals.

## Antimicrobial resistance surveillance in people and animals

Antimicrobial resistance surveillance began in Nepal in 1999 by the National Public Health Laboratory (NPHL) as a focal point for AMR surveillance with technical assistance from Bangladesh (17). Surveillance on AMR for animal pathogens did not commence, however, until 2011 with co-ordination between NPHL and various veterinary laboratories (17). This collaboration did not produce any fruitful groundbreaking data. AMR resistance surveillance has benefited from funding by the Fleming fund as “Tackling Antimicrobial Resistance in Nepal” (https://www.lstmed.ac.uk/research/centres-and-units/capacity-research-unit-cru/our-projects/antimicrobial-resistance projects/antimicrobial-resistance). A draft strategic framework on AMR/AMD has been presented to the Ministry of Health (MoH) for approval (18, 19) and National Antibiotics Treatment Guidelines have already been endorsed by the Ministry (20).

## Antibiotic Resistance Status

Antibiotics, at USD 1 billion/year, account for about 30% of the total value of medicine imports (15). Some 365 brands of antibiotics are available on the Nepalese market and antibiotics are produced within the country by about 50 companies. Antimicrobials are often imported as feed additives or feed supplements as these are free of or pay a low rate of customs duty and registration charges (15). Poultry products are used without consideration of withdrawal periods even by commercial producers (5). There is extreme abuse of antibiotics in cattle, pigs and poultry as growth promoters and to minimize production losses (5, 15). Sub-therapeutic doses used for growth promotion and disease prevention are likely to create resistant microbes that are transmissible to humans (21). Safe and judicious use of antibiotics on dairy farms is still not practised and the risk to public health of residues in meat and milk is high (Table 1).

**Table 1:** Antimicrobial residues in meat and milk.

**Antimicrobial agent**	**Sample**	**Resistance (%)**	**Source**
Tetracycline	Chicken (*n* = 50)	10	(22)
Penicillin	Milk (*n* = 98)	13.2	(23)
Penicillin	Chicken (*n* = 80)	40	(24)
Sulphonamides, penicillin Qualitative (meaning)	Milk (*n* = 140)	23	(25)
Sulphonamides, penicillin	Milk (*n* = 150)	17.3	(26)
Sulphonamides	Chicken (*n* = 25)	96	(27)
Sulphonamides	Milk (*n* = 50)	20	(27)

### Antimicrobial Resistance in Human Health Sector

Thirteen studies of antibiotic prescribing pattern in Nepal showed most patients were unnecessarily prescribed more than one antibiotic concurrently without bacterial confirmation or susceptibility testing (16, 28). A reported 10–42% of patients were prescribed antibiotic for both therapeutic and prophylactic purposes. One study conducted in 1998 showed that 68% of drugs prescribed and 70% of prescriptions for respiratory infections were antimicrobials (29). The low number of physicians to patients (1:1724) indicates that most Nepalese must rely on health assistants and pharmacists as the primary source of allopathic health care (30). Some 97% of medicines distributed by these two outlets for routine symptoms such as diarrhoea are antimicrobials (31) and thus provide a favourable environment for development of antimicrobial resistance. Walson et al. (29) reported that the higher prevalence of multidrug-resistant bacterial strains in their remote study village was due to lack of contact with health posts and hospitals. In rural areas water sources are used for various purposes such as drinking, bathing, laundering, and irrigation which enhances the probability of shared bacterial strains among these populations (29). Significantly higher proportions of multidrug resistant bacterial strains close to health care centres and hospitals have been reported due to intense exposure to antibiotics (32). Antibiotics used against bacteria in urine, pus and blood of infected patients in several Nepali hospitals were effective in curing only 50% of cases whereas the other 50% showed no response (33). Recently it has been shown that most antibiotics intended to cure people are becoming less effective due to development of partial resistance by bacteria (34). Amoxicillin, ciprofloxacin, and norfloxacin used against *E. coli* are effective in < 40% of infected people and effective in < 20% in pneumonia caused by *Klebsiella* spp. (1).

Self-medication is common in Nepal (15) and most people do not comply with the treatment prescribed by the physician (1, 15). Most patients fail to follow a full course of treatment and usually stop taking medicines after 2–3 days when symptoms start to subside. They then store the leftover medicines for future self-medication, thereby prolonging the duration of illness and speeding up the rate of development of resistance (16, 35, 36). In addition, antibiotics are easily available from pharmacies without prescriptions (15). Physicians are also responsible for the growing burden of AMR: even highly unsafe drugs categorized as “Group A” and “Group B” are prescribed in an irrational way, thus facilitating the possibility of development of resistance (1). It is known that some practitioners prescribe broad spectrum antibiotics even when the problem can be overcome by a narrow spectrum drug. It is not unknown for patients to be prescribed the wrong medicines which can exacerbate the condition (15). Antibiotics are prescribed for colds, coughs and diarrhoea that are likely to heal following a simple course of supportive treatment (16). Health care workers commonly dispense incorrect doses and provide incorrect guidance on how medicines should be taken (16).

Pharmaceutical companies with vested interests offer incentives to physicians to prescribe “their” drugs (19). Some physicians attempt to deprive people of their money by prescribing too many drugs, e.g., ciprofloxacin plus other antibiotics, with no consideration of product compatibility. In some hospitals and in intensive care units overuse of antibiotics and a high density of patients contribute to the increased pace of antimicrobial resistance (37). Some 74.1% of hospitals in Nepal do not have waste water treatment facilities (38). The effluents from hospital could directly reach the human body through contaminated drinking water, as most people in Nepal have poor sanitation and hygienic status. Similarly, there is a high risk of spread of resistant microbes when patients are discharged to continue medication at home and are in direct contact with other people in the household (39). In addition, due to poor infection control practices, and lack of quality compliance and monitoring, nosocomial infections with highly resistant bacterial pathogens are rapidly increasing (Table 2).

**Table 2:** Some selected results of research on AMR in the Human Health Sector.

**Location**	**Date**	**Organism isolated**	**Resistance (%)**	**Source**
Kathmandu: Kanti Hospital (*n* = 538)	2008	*E. coli*	Norfloxacin (64); Cotromoxazole (77); Nalidixic acid (78); Cephalexin (97)	(40)
Pokhara: school children (*n* = 32)	2008	Methicillin-resistant *Staphylococcus aureus* (MRSA)	Cloxacillin (68.70); Ofloxacin (40.60); Tetracycline (15.60); Erythromycin (9.40); Ciprofloxacin (6.20); Vancomycin (3.1)	(41)
Kathmandu: National Public Health Laboratory (*n* = 53)	2005	*Vibrio cholerae*	Nalidixic acid (100); Cotrimoxazole (100); Furazolidone (85)	(42)
Kathmandu: National Public Health Laboratory (*n* = 57)	2008–2009?	*Vibrio cholerae*	Nalidixic acid (100); Cotrimoxazole (100); Furazolidone (100); Ampicillin (26); Erythromycin (32)	(43)
Kathmandu: Medical College (*n* = 136)	2011	*E. coli*	Cotrimoxazole (39); Ofloxacin (60); Norfloxacin (59); Ciprofloxacin (57)	(44)
Koshi: Zonal Hospital (*n* = 55)	2012	*Klebsiella pneumoniae*	Ciprofloxacin (33); Cotrimoxazole (85); Nalidixic acid (87); Nitrofurantoin (5)	(45)
Western Regional Hospital (*n* = 6)	2012	*E. coli*	Azithromycin (75); Cefexime (67); Nalidixic acid (83); Ampicillin (0); Ceftriaxone (0)	(45)
Kathmandu: Children's Hospital (*n* = 24)	2012	*Shigella* sp.	Ampicillin (50); Nalidixic acid (54.20); Norfloxacin (8.30); Chloramphenicol (20.80); Cotromoxazole (50); Gentamicin (8.30); Ciprofloxacin (8.30); Tetracycline (41.70); Ofloxacin (8.30); Amikacin (12.50); Ceftazidime (25); Cefotaxime (33.33)	(46)
Kathmandu: Medical College (*n* = 208)	2012	*E. coli*	Ofloxacin (37); Cotrimoxazole (48); Amoxycillin (33)	(47)
Accham, Baitadi, and Doti Districts (*n* = 27)	2013	*Vibrio cholerae*	Nalidixic acid (100); Cotrimoxazole (100)	(48)
Kathmandu: Medical College (*n* = 208)	2013	*E. coli*	Ofloxacin (60); Ciprofloxacin (49); Cephalexin (51); Norfloxacin (60); Amoxycillin (83)	(49)
Pokhara; Manipal Teaching Hospital (*n* = 40)	2004–2010	*Neisseria gonorrhea*	Penicillin (67.5); Tetracycline (45); Ciprofloxacin (42.)	(49)
Birgung: National Medical College and Teaching Hospital (*n* = 30)	2009	*Neisseria gonorrhea*	Penicillin (0); Tetracycline (33.3); Ciprofloxacin (20); Ceftriaxone (0)	(50)
Kathmandu Valley	1998–2002	*Salmonella typhi*; *Salmonella paratyphi* A	Ciprofloxacin (2); Ciprofloxacin (4)	(51)
Kathmandu Valley	2008–2011	*Salmonella typhi*; *Salmonella paratyphi* A	Ciprofloxacin (11); Ciprofloxacin (14)	(51)
Kathmandu Valley	2008–2011	*Salmonella paratyphi* A	Nalidixic acid (91)	(51)
Neonatal sepsis (resistance range 50–100)	2015	*Staphylococcus aureus*; *Klebsiella pneumoniae*; *Pseudomonas* spp.; *Acinetobacter* sp.; Enterobacteriaceae	Ampicillin; Cefotaxime; Ceftriaxone; Imipenem; Ceftazidime; Piperacillin	(1)
Patan: Academy of Health Science	2012–2016	*E. coli* 60 (68); *Klebsiella* sp.; 15 (17); *Proteus* sp. 7 (8); *Enterococcus* sp.	Ampicillin 51 (85); Ofloxacin 49 (82); Cefotaxime 45 (75); Gentamicin 17 (28); Amikacin 2 (3)	(52)
Eastern Nepal: Tertiary Care Hospital (*n* = 52)	2013–2014	Coagulase -ve *Staphylococci (CoNS)*	Ampicillin (80); Cefoxitin (58); Ceftriaxone (58)	(53)
Pokhara: Manipal Teaching Hospital (*n* = 400)	2012–2013	Methicillin-resistant *Staphylococcus aureus* (MRSA)	Penicillin 139 (100); Erythromycin 10 (73.40); Ciprofloxacin112 (80.50); Cotrimoxazole 93 (66.90); Cefazoline 41 (29.50); Gentamicin 66 (47.50); Clindamycin 15 (10.80); Amikacin 14 (10.00); Tetracycline 10 (7.20); Vancomycin 0 (0)	(54)

### Antimicrobial Resistance in Animal Health Sector

**Figure 2:**
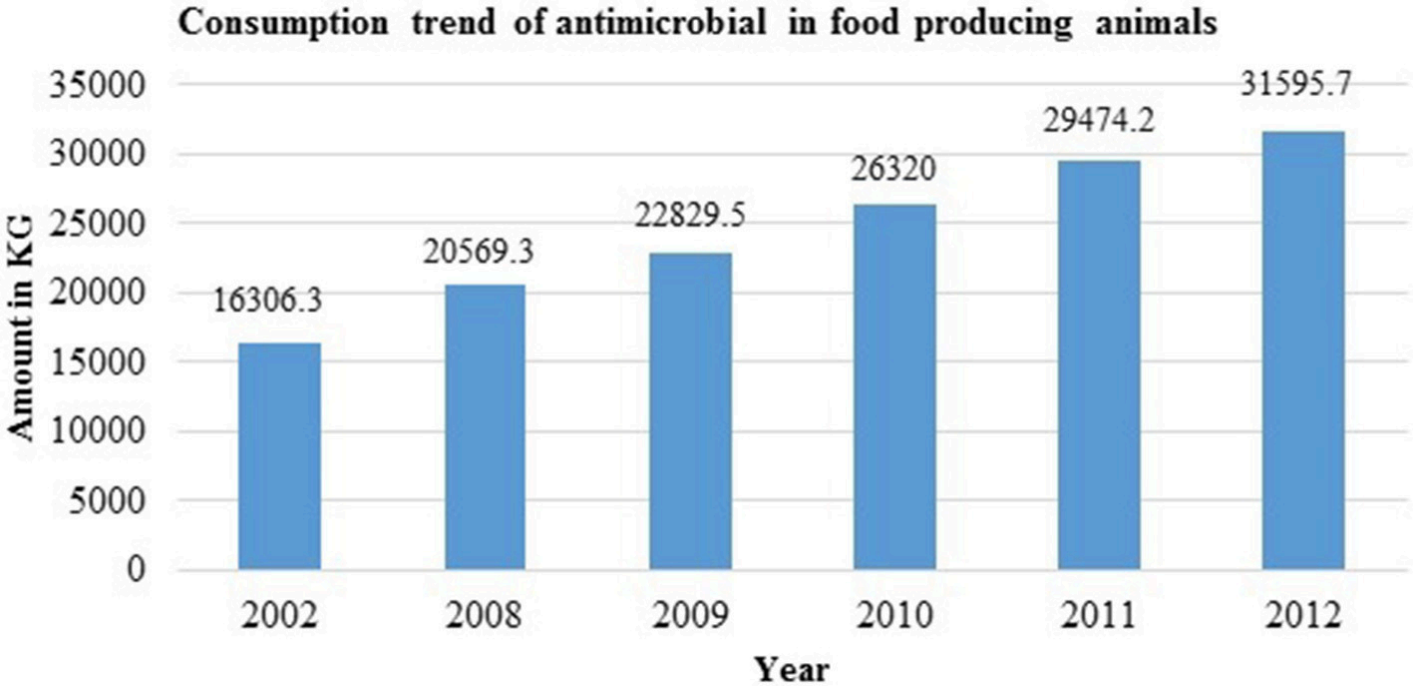
Consumption trend of antimicrobials in food producing animals [Source: Bhandari and Singh (55); Khatiwada (56)].

**Figure 3:**
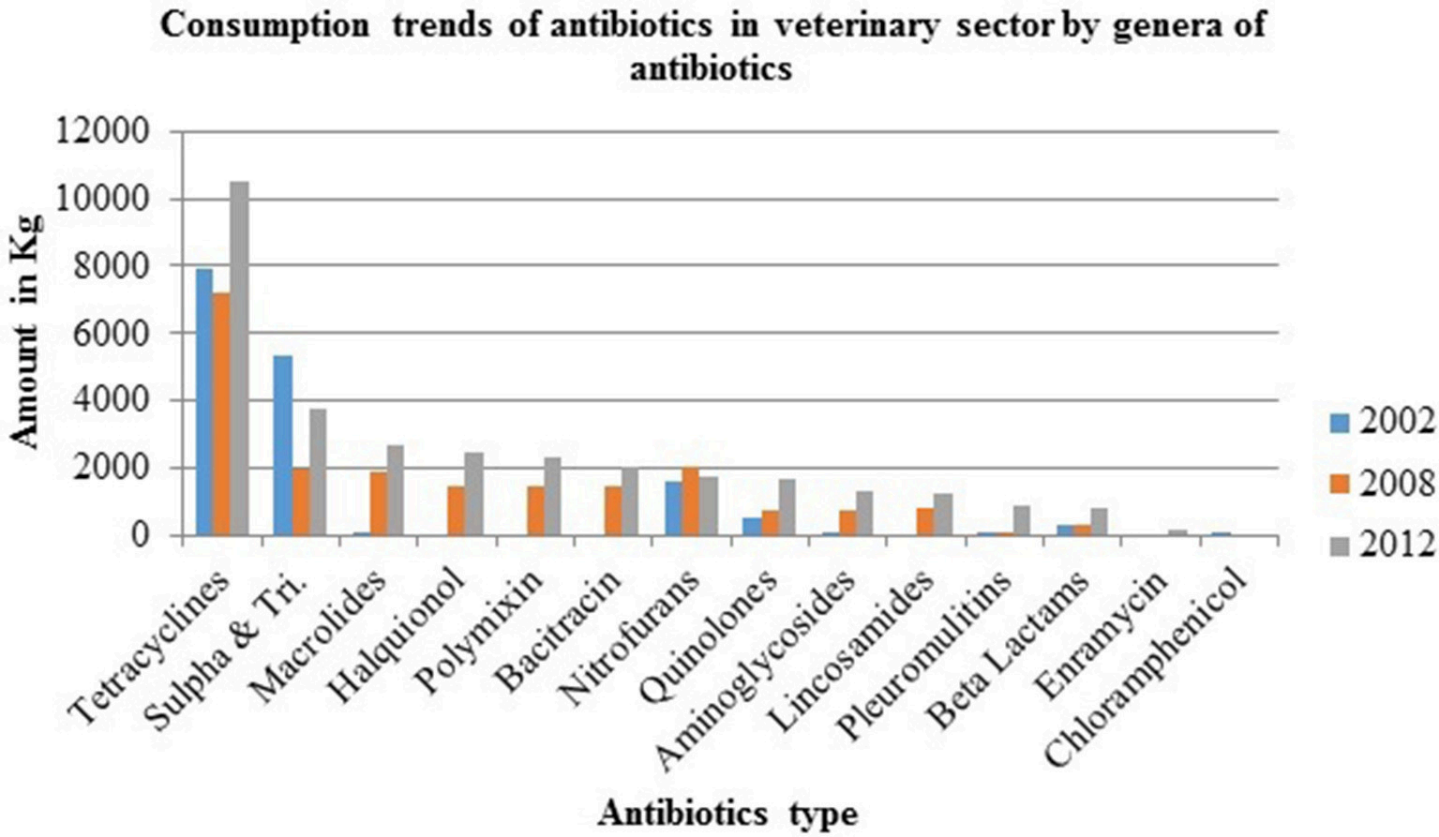
Consumption trends of antibiotics in veterinary sector by type [Source: (Bhandari and Singh (55); Khatiwada (56)].

A survey on distributors of veterinary medicines and feed supplements in 2003 in six Nepali districts reported annual sales of USD 6.7 million. Some 13% of total veterinary expenditure was on antibiotics whose sales rose by 50% between 2008 and 2012 (1) (Figure 2). Over 70% of veterinary drug sales were obtained from para-professionals or retail outlets (which do not have proper storage facilities and whose staff usually have no veterinary training) and not prescribed by veterinary professionals (51). Tetracycline, enrofloxacin, neomycin-doxycycline, levofloxacin, colistin, and tylosin are the top seven antibiotics consumed in Nepal (5, 14, 55) (Figure 3) with ampicillin, amoxicillin, ceftriaxone, and gentamicin being the most inappropriately prescribed medicines (28). A study by Acharya showed that 35.1% of drug sellers practised self-prescription whereas 40.4% of dispensed antibiotics were based on prescription by veterinarians (14). Similarly, 71% of veterinary drugs sold in Nepal were based on prescription by paraprofessionals and drug retailers (56). One study that examined prescription behavior by drug dispensers in Biratnagar, Kathmandu, Chitwan, Pokhara, and Surkhet (the main hotspots for drug sales in Nepal) found around 46% of veterinary drugs were sold under self-prescription (5) and 12% were based on farmer demand (5). Retailers and distributors do not have adequate knowledge on effective dosage and the possible side effects of veterinary drugs (55). Several farmers administer antibiotics usually in consultation with neighbours and their own previous experience rather than qualified veterinarians/ practitioners (5, 15). Most farmers are not aware of antimicrobial residue and drug withdrawal periods (5). Farmers themselves use antibiotics to compensate poor farm sanitation and hygiene (5, 14, 57). This ignorance of drug withdrawal periods and its negative impact on animal and human health combined with long term use leads to animal products arriving on the market with residues above the permitted (MRL) which increases antimicrobial resistance(5, 15). Resistant microbes in animals are transmitted to humans (58, 59) either through the food chain or via direct or indirect contact with animals.

According to the Department of Drug Administration (DDA), veterinary drugs worth 70 million rupees were imported by Nepal in 2006 when domestic production was 14 million rupees and drugs to the value of 155 million rupees were sold (60). There is an abundance of high cost, poor quality drugs on the market (60) but administration to animals does not consider veterinary ethics (5). There are inadequate regulations and guidelines for the use of veterinary drugs and there is an absolute lack of antimicrobial resistance surveillance in the veterinary field (61). Thus, in the absence of effective veterinary drug regulation and co-ordination among authorities, the veterinary antimicrobial market is unorganized and haphazard (5). Analyses of Antibiotic Sensitivity Test reports the Central Veterinary Laboratory have shown increasing resistance by micro-organisms to a widening range of antibiotics (61).

The poultry sector, in which the use of antibiotics has increased many fold in recent times and in which antibiotics are used to reduce loss from morbidity and mortality of chicks (15), is notorious for its increasing antimicrobial resistance (61). A large body of evidence shows that the use of antimicrobial agents in food animals is strongly and clearly linked to antimicrobial resistance among bacteria isolated from humans. Some very common examples are AMR in *Salmonella, Campylobacter* and *Escherichia* (36). Resistant strains of *Escherichia coli* in young children of the rural village of Barcelona have been transmitted from poultry and pork given sub-therapeutic level of antibiotics as growth promoters (62). There has, however, been a very considerable increase in antibiotic use in all food-producing animals in recent years from 2002 to 2012 (Figures 2, 3) (55, 56).

In large annual sampling schemes (*n* = >1000) isolates including *E. coli, Staphylococcus aureus, Streptococcus, Micrococcus*, and *Pasteurella* were found to be highly resistant to enrofloxacin, amoxicillin, and colistin sulphate but less sensitive to tetracycline, gentamicin and levofloxacin (Table 3) and highly sensitive to non-conventional drugs (in the veterinary field in the context of Nepal) such as cefoxitine, ceftriaxone, chloramphenicol, and ciprofloxacin (71). *Pseudomonas* was commonly reported in water and in poultry and other meat samples and was highly resistant to conventional drugs such as enrofloxacin, amoxicillin, and colistin sulphate (70).

**Table 3:** Some selected results of research on AMR in the Animal Health Sector.

**Location**	**Year**	**Organism isolated**	**Resistance (%)**	**Source**
Chitwan: poultry liver, gizzard and breast muscles (*n* = 225)	2015	*E. coli*	Furazolidone (98.1); Tetracycline (88.9); Cephalexin (79.6)	(5)
Chitwan: poultry liver, gizzard and breast muscles (*n* = 225)	2015	*Staphylococcus*	Furazolidone (91.7); Cotrimoxazole (62.5); Enrofloxacin (54.2); Gentamicin (14.6); Cephalexin (12.5); Tetracycline (10.4)	(5)
Chitwan: poultry liver, gizzard and breast muscles (*n* = 225)	2015	*Salmonella*	Furazolidone (82.1); Tetracycline (32.1); Cephalexin (28.6); Cotrimoxazole(17.9); Enrofloxacin (14.3); Colistin (10.7)	(5)
Chitwan: 7 hatcheries (*n* = 140)	2013	*E. coli*	Amoxicillin (93); Tetracycline (86); Enrofloxacin (50); Gentamicin (43); Ciprofloxacin (36)	(63)
Chitwan: minced buffalo meat (*n* = 63)	2012	*E. coli*	Cotromoxazole (79.60); Enrofloxacin (68.50); Colistin (25.90); Cephalexin (20.40); Nitrofurantoin (8);	(64)
Biratnagar: Regional Veterinary Laboratory	2012	*E. coli*; *Staphylococcus* spp.; *Streptococcu*s spp.; *Klebsiell*a spp.; *Pseudomonas* spp.; *Enterobacter* spp.	Cefotaxime (100); Chloramphenicol (67); Tetracycline (54); Gentamicin (44); Ciprofloxacin (35); Enrofloxacin (35)	(65)
Pokhara: dairy farms (*n* = 400 from 100 animals)	2013	Methicillin-resistant *Staphylococcus aureus* (MRSA)	Cefoxitin 45 (37.82); Ceftriaxone 10 (8.40); Ciprofloxacin 3 (2.52); Gentamicin 6 (5.04); Tetracycline 12 (10.08); Cotromoxazole 32 (26.89)	(66)
Chitwan: Mangalpur and Rampur VDCs (*n* = 400 from 100 animals)	2015	Methicillin-resistant *Staphylococcus aureus* (MRSA)	Cefotaxime (18.20); Gentamicin (2.90); Amoxyclav (54.70); Clindamycin (12.40); Norfloxacin (1.50); Tetracycline (5.80); Cefotaxime (46.00);	(67)
Kathmandu: milk samples (*n* = 206)	2012–2013	*E. coli*	Gentiamicin (15); Tetracycline (20); Enrofloxacin (70); Ampicillin (59); Amoxycillin (65); Cephalexin (70); Doxycycline (92); Cotrimoxazole (97); Neomycin (93); Ciprofloxacin (13); Azithromycin (95); Colistin (69)	(68)
Kathmandu: milk samples (*n* = 213)	2013	*Staphylococcus* spp.	Gentiamicin (21); Tetracycline (20); Enrofloxacin (94); Ampicillin (42); Amoxycillin (70); Cephalexin (70); Doxycycline (91); Cotrimoxazole (97); Neomycin (95); Ciprofloxacin (25); Azithromycin (96); Colistin (67)	(68)
Kathmandu: milk samples (*n* = 13)		*Streptococcus* spp.	Gentiamicin (69); Tetracycline (69); Enrofloxacin (85); Ampicillin (77); Amoxycillin (–); Cephalexin (69); Doxycycline (92); Cotrimoxazole (–); Neomycin (–); Ciprofloxacin (77); Azithromycin (–); Colistin (85)	(68)
Kathmandu: milk samples (*n* = 15)	2013	*Bacillus* spp.	Gentiamicin (47); Tetracycline (53); Enrofloxacin (67); Ampicillin (60); Amoxycillin (93); Cephalexin (87); Doxycycline (92); Cotrimoxazole (–); Neomycin (–); Ciprofloxacin (–); Azithromycin (40); Colistin (87)	(68)
National Avian Laboratory: poultry postmortem samples (*n* = 63)	2012	Various microorganisms	Tetracycline (33.33); Chloramphenicol (20); Ciprofloxacin (0); Gentamicin (41.20); Amikacin (0); Levofloxacin (17.70); Cephalexin (0); Ceftriaxone (0); Norfloxacin (33.33); Cotromoxazole (75)	(69)
Kathmandu: milk samples (*n* = 127)	2015–2016	*E. coli*	Gentamicin (1.8); Enrofloxacin (0); Cephalexin(2); Amoxycillin(63); Tetracycline (3); Ofloxacin(66); Ampicillin(82); Azithromycin(6); Colistin(0); Levofloxacin(10); Norfloxacin(87); Erythromycin(85); Ciprofloxacin(8); Amikacin(0); Chloramphenicol (26)	(70)
Kathmandu: milk samples (*n* = 134)	2015–2016	*Staphylococcus aureus*	Gentamicin (6); Enrofloxacin (19); Cephalexin(6); Amoxycillin(76); Tetracycline (7); Ofloxacin(–); Ampicillin(68); Azithromycin(98); Colistin(41); Levofloxacin(68); Norfloxacin(90); Erythromycin(80); Ciprofloxacin(30); Amikacin(0); Chloramphenicol (39)	(70)
Kathmandu: milk samples (*n* = 4)	2015–2016	*Bacillus* spp.	Gentamicin (7); Enrofloxacin (15); Cephalexin(0); Amoxycillin(45); Tetracycline (3); Ofloxacin(50); Ampicillin(47); Azithromycin(9); Colistin(12); Levofloxacin(14); Norfloxacin(–); Erythromycin (–); Ciprofloxacin (12); Amikacin(50); Chloramphenicol (4.00)	(70)
Kathmandu: milk samples (*n* = 8)	2015–2016	*Streptococcus* spp.	Gentamicin (33); Enrofloxacin (75); Cephalexin(14); Amoxycillin(25); Tetracycline (44); Ofloxacin(50); Ampicillin(33); Azithromycin(50); Colistin (–); Levofloxacin (–); Norfloxacin(0); Erythromycin (–); Ciprofloxacin (–); Amikacin(–); Chloramphenicol (16)	(70)

Antibiotics are widely used as a growth promoter and to reduce mortality in cattle, pigs, and poultry (5). Sub-therapeutics doses of antibiotics are likely to create resistant microbes that are transmissible to humans (21). Safe and judicious use of antibiotics on dairy farms is rarely practised (14, 15, 56). Antibiotics such as chlortetracycline (CTC), bacitracin methylene disalicylate, tylosine tartarate, lincomycin, neomycin, and doxycycline are widely used in poultry feed as additives or growth promoters (5) (Table 4).

**Table 4:** Antimicrobials used in feed as growth promoter in poultry feed in Nepal.

**SN**	**Antimicrobials**	**Mixing rate**
1	Bacitracin methylene	500 gm to 1 kg/ton
2	Neomycin	500 gm to 1 kg/ton
3	Doxycycline	500 gm to 1 kg/ton
4	Furazolidone	500 gm to 1 kg/ton
5	Chlortetracycline	500 gm to 1 kg/ton
6	Tylosine	500 gm/ton
7	Lincomycin	250–500 gm/ton
8	Colistin sulphate + Doxycycline	500 gm/ton
9	Tetracycline + Tiamutin	1–2 kg/ton
10	Bacitracin +Lincomycin+Colistin sulphate	250–500 gm/ton

*[Source: Ramdam (5)]*.

Antimicrobials are imported as feed additives and feed supplements in order to avoid or reduce the amount of customs duty and registration (15). Many products are used even by commercial farmers with no consideration for minimal withdrawal periods (5). This failure to follow the recommended withdrawal period (5, 15, 72) and easy availability of drugs over the counter and farmers' self-prescription (5, 16, 56) might have contributed to increasing drug resistance in Nepal. Apart from this, availability of substandard and counterfeit drugs, lack of proper advice on scientific husbandry practices with poor hygiene and sanitation, overuse of antimicrobials, lack of awareness and observance of drug withdrawal periods are some of the most important factors in the growing AMR burden in Nepal (1, 5).

**Figure 4:**
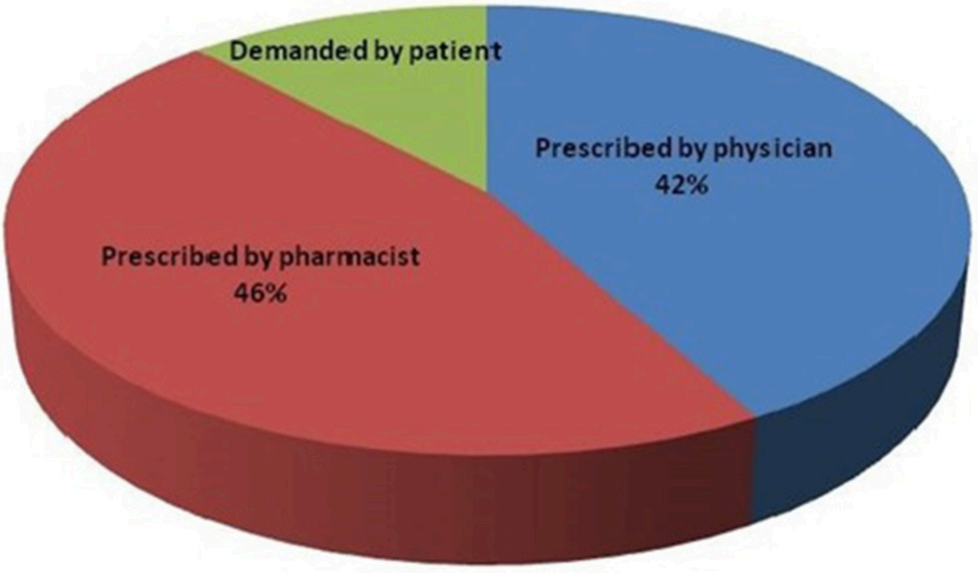
Basis of antibiotics sales by Veterinary practitioners' in Nepal [Source: Ramdam (5)].

Self-prescription, over-prescription, under-prescription, empirical antibiotics therapy and irrational prescription of powerful antibiotics intended for rapid cure have all contributed to antibiotic resistance. Veterinarians fail to provide correct doses and the proper duration of treatment (73). There is no laboratory strategic plan to provide guidance and governance to national laboratories. Information about antimicrobial resistance is thus not properly communicated to health workers and paraprofessionals who prescribe medicines. A recent study carried out under Global Antibiotics Resistance Partnership (GARP) funded research demonstrated that 46% of veterinary drugs were sold under self-prescription and about 12% based on farmers' demand (Figure 4) (5).

## Policy and legal standards

The Drugs Act 2035 (74) is the only legislative instrument designed to regulate the use of both allopathic and herbal drugs in Nepal. The main aims of the Act are to prohibit misuse or abuse of drugs and allied pharmaceutical materials, to prohibit false and misleading information related to the efficacy and use of drugs and to regulate and control production, marketing, distribution, export-import, storage and the use of drugs that are not efficacious, not of standard quality and not safe for use by people. The DDA is the sole authority responsible for regulating allopathic, ayurvedic and homeopathic drugs in Nepal in addition to regulating veterinary drugs. The Veterinary Standard and Drug Administration Office (VSDAO), under the Directorate of Animal Health is effectively a sinecure as a Veterinary Drugs Act is yet to be promulgated. A bill has been drafted and is in the pipeline for approval from cabinet and parliament but conflict among stakeholders has greatly slowed progress although it does regulate the import of veterinary vaccines (15). Designated Veterinary Inspectors in each district do little other than inspect drug stores and monitor their functioning. They are not authorized to institute legal proceedings which can only be done following a tedious and lengthy process by the DDA. Similarly the VSDAO can take no action itself (15). At present, veterinary drugs and biological are jointly inspected by DDA, MoH and VSDAO. Issues around drug residues and resistance are addressed by the Veterinary Public Health Office (VPHO), the Central Veterinary Laboratory (CVL) and VSDAO. VSDAO in collaboration with DDA and the Directorate of Livestock Training and Extension does provide training or veterinary drug sellers (22).

The Ministry of Health and Population (MoHP) has attempted to address the issue of AMR by the release of a National Antibiotics Treatment Guidelines in 2014. The Ministry of Livestock Development (MoLD) has promoted a policy of zero antibiotics in feed supplements, with a hope to prohibit the use of antimicrobials as growth promoters and to implement stringent awareness and orientation programme to stop the use of antimicrobials at sub-therapeutic doses. VSDAO and VPHO are highlighting the problems with continuous awareness programmes and monitor antimicrobial residues in agricultural products and livestock products (milk, meat and eggs). The Department of Livestock Services (DLS) and MoLD attempt to ensure strict compliance of withdrawal periods in meat, milk and eggs. A national multi-sectoral committee has been given responsibility for implementation of the National Action Plan for Antimicrobial Resistance (NAP-AMR).

DDA and DLS are strict in the registration of new drugs manufactured in or imported to Nepal and in regulating drugs in the country. DLS has restricted the import of feed supplements containing antibiotics since 2016. Recently an important initiation has been taken by the (MoHP) to actively deal with antimicrobial resistance. The National Public Health Laboratory (NPHL), with technical support from the World Health Organization (WHO) has been conducting laboratory-based antimicrobial resistance surveillance since 2005 and has demonstrated the dreadful resistance of microbes to common antimicrobials and the national drug policy was amended by addition of a clause on “Prudent use of antibiotics.” Laboratories under MoLD, DLS have been involved in AMR surveillance since 2011 in collaboration with NPHL.

## Preventive and control measures against antimicrobial resistance

Government of Nepal has to strictly implement a national action plan on AMR. This should include strategies and policies to promote good husbandry practices, nationwide AMR surveillance program, and to raise awareness among producers and consumers on issues of AMR. Moreover, irrational use of antibiotics and the illegal import of medicines need to be strictly controlled. Equal emphasis should be given to reduce antimicrobial use as far as possible, and immunization and vaccination program to prevent and control the infectious diseases. A coordinated one health surveillance initiatives on antimicrobial use (AMU) and antimicrobial resistance (AMR)- involving various players like government agencies, medical personnel, veterinarians, livestock producers/farmers, is needed. Healthcare professionals should be trained on AMR issues, raising awareness among public and farmers on harmful effects of drugs to their bodies and hazards of development of antimicrobial resistance. Users of antimicrobials need to be made aware of harmful effects of unnecessarily prescribed drugs and its effect on increase in problem of antibiotic resistance. Healthcare professionals should be restricted to the use of antibiotics only when needed. There should be strong collaborative research on the development of strategies to minimize antimicrobial resistance either by optimal use of antibiotics or by other novel approaches.

The educational system should include modules on antimicrobial resistance and reduce use of antimicrobials in hospitals and at community level as well as its use in agriculture. Curricula and treatment guidelines should be regularly revised. Antibiotic stewardship programmes need to be implemented. Strong commitment from all stakeholders is required including especially political commitment. Antimicrobial stewardship should be driven by public-private partnership approaches with government legislating, regulating and taking legal action on rationale use of antibiotics based on public interest. Establishment of a surveillance programme for AMR and creating awareness among health care professionals regarding AMR and prudent drug use in Nepal are mandatory. We all should be very conscious on use of antibiotics, follow sanitary and hygienic practices, follow good hygiene and limit the use of antibiotics as growth promoter in animal and agriculture. Last but not least, concerned stakeholders should think conscientiously whether it is a good decision, whether antibiotic is necessary and whether it is safe for human and animals.

## Author Contributions

KA conceived the original paper and provided a rough draft. RW checked the paper for consistency, corrected the language and checked all references for consistency and accuracy.

### Conflict of Interest Statement

The authors declare that the research was conducted in the absence of any commercial or financial relationships that could be construed as a potential conflict of interest.
